# Neurologist practice patterns in treatment of muscle cramps in Canada

**DOI:** 10.1186/1757-1146-6-2

**Published:** 2013-01-09

**Authors:** Mary Jane Lim Fat, Seint Kokokyi, Hans Dieter Katzberg

**Affiliations:** 1 Toronto General Hospital / University Health Network, 200 Elizabeth Street, Toronto, ON, M5G 2C4, Canada; 2 University of Toronto, Faculty of Medicine, 1 King’s College Circle, Toronto, ON, M5S 1A8, Canada

**Keywords:** Muscle cramps, Baclofen, Quinine, Practice parameters

## Abstract

Recently an article provided patient perspectives on therapies and perceived effectiveness in preventing muscle cramps. However, there are few studies evaluating physicians’ point of view in the management of this common symptom. In our study, we studied physician practice patterns in the treatment of muscle cramps by surveying a group of neurologists in Canada. We demonstrated that most physicians use a combination of pharmacological and non-pharmacological methods in treating muscle cramps. The most commonly used medications are baclofen, quinine and gabapentin, of which baclofen and quinine were reported to be the most tolerated.

## 

We read with great interest the article written by Blyton *et al.* evaluating patient experience of treatments for muscle cramps [1]. In 2010, we presented a review of existing literature on the treatment of muscle cramps [2], however, there have been few publications that bridge the gap between evidence-based medicine and clinical practice as this article does. In this commentary, we present additional data on the practice patterns of a group of Canadian neurologists in the management of muscle cramps in an effort to further connect this gap.

With the approval of University Health Network Research Ethics Board, two hundred and eighty four physicians were sent an invitation with informed consent to complete the survey. A total of 62 physicians responded of which 8 declined to participate. Physician experience ranged from those starting practice to over 30 years experience. Half of the physicians identified themselves as general neurologists and 25% as neuromuscular specialists. Four physicians among the fifty-four responders (7.4%) prescribed no treatments for muscle cramps, while 38 physicians (70.4%) used a combination of both pharmacological and non-pharmacological treatments. An equal number of physicians (n = 6, 11.1%) prescribed only pharmacological or only non-pharmacological treatments. Forty-three percent of neurologists used multiple pharmacological treatments and 70.4% used multiple non-pharmacological agents. Table 1 shows the pharmacological and non-pharmacological agents used by neurologists in the treatment of muscle cramps. The most common non-pharmacological methods included hydration and stretching and the top 3 pharmacological agents were baclofen, quinine sulphate and gabapentin. Baclofen and quinine derivatives were identified equally as the best-tolerated medications.

**Table 1:** Non-pharmacological and pharmacological agents used by neurologists (n = 54) in the treatment of muscle cramps, as well as the percentage of physicians who used each particular agent

**Factor**	Number of physicians prescribed, n (%)*	Number of physicians rating agent as most tolerable, n (%)*
Non-Pharmacological Treatment		
Hydration	36 (66.7)	--
Stretching	24 (44.4)	--
Massage Therapy	21 (38.9)	--
Herbal remedies	4 (7.7)	--
Pharmacological Treatment		
Baclofen	29 (53.7)	11 (20.4)
Quinine and its derivatives	27 (50.0)	11 (20.4)
Gabapentin	27 (50.0)	5 (9.3)
Carbamazepine	15 (27.7)	0 (0.0)
Verapamil	12 (22.2)	0 (0.0)
Vitamin B	9 (16.7)	13 (16.7)
Phenytoin	9 (16.7)	0 (0.0)
Diltiazem	5 (9.2)	1 (1.9)
Levetiracetam	4 (7.4)	0 (0.0)
Vitamin E	3 (5.5)	6 (11.1)
Oxycarbazepine	1 (1.9)	0 (0.0)

* Percent numbers do not add up to 100 due to multiple responses.

Prescription-grade quinine appears to be one of the most effective medications for cramps according to neurologists in our study and “quite effective-100% effective” in 14/18 patients in the Blyton study. Cramp away or crampeze in contrast, which contain much lower amounts of quinine than the 300 mg of quinine used in most trials, is found to be useless or of little help most of the time. This type of data is important to patients and physicians, as prescription guidelines for quinine are country specific. The FDA (USA) and TGA (Australia) have banned the marketing of quinine derivatives for muscle cramps [3,4]. Although the intention of our questionnaire was not to identify adverse events associated with quinine, it does show that prescription-strength quinine continues to be prescribed frequently and with good tolerability in Canada. Other published reviews have supported the use of quinine derivatives for short-term administration (under 60 days) [5] and in the physician-supervised treatment of disabling cramps [2].

Both studies reported the frequent use of non-pharmacological agents in the management of muscle cramps, most commonly stretching and hydration. Although these interventions had limited perceived efficacy according to patients, the low risk makes them good options as first-line recommendations. A comprehensive list of over-the-counter and homeopathic treatments was presented in the Bylton paper. Although magnesium is used frequently by patients, we agree that it has a limited role in treating muscle cramps outside the setting of pregnancy. None of the physicians in our survey used magnesium and our review found no evidence for their use in idiopathic muscle cramps [2]. Information gathered in our survey also supplements the Blyton article, which presented limited data on prescriptions medications. Medications other than quinine included baclofen (used most frequently and with best tolerability), gabapentin, calcium channel blockers, clonazepam, diazepam, and mexilitine. Of all these medications, only calcium channel blockers have modest evidence for use in cramps [2].

There are methodology limitations in both questionnaire-based studies, including recall bias, limited information on side effects and etiologies of cramps, including medications [6].

Although caution needs to be used in interpreting these results, we hope that they will provide information relevant to clinical practice as serve as a catalyst for additional case–control and treatment trials for muscle cramps.

Our study shows that a combination of pharmacological and non-pharmacological methods was employed in treating muscle cramps. Moreover, physicians turn to baclofen, quinine and its derivatives, and gabapentin for pharmacological agents.

## Abbreviations

FDA: Food and Drug Administration; TGA: Therapeutics Good Association.

## Competing interests

Dr. Lim Fat has no financial or non-financial competing interests.

Miss Kokokyi has no financial or non-financial competing interests.

Dr. Katzberg has no financial or non-financial competing interests.

## Authors’ contributions

MJLF participated in the design of the study, and faxed surveys to neurologists. SK participated in the coordination of the study, mailed surveys to non-responders and reconciled all completed surveys. HDK participated in the design of the study, e-mailed surveys to non-responders and supervised the study. All authors performed statistical analysis, helped to draft the manuscript, read and approved the final manuscript.
